# Laryngology and Rhinology

**Published:** 1902-12

**Authors:** P.Watson Williams


### LARYNGOLOGY AND RHINOLOGY.

**Direct endoscopy of the trachea and bronchi.** Direct inspection of the bladder, and of the œsophagus and stomach, has become generally recognised as affording valuable aids to the diagnosis and treatment of diseased conditions of these cavities; but till very recently direct endoscopy of the trachea has presented insuperable difficulties. By Killian's method[1] of passing a long straight tube of sufficient length and a width small enough to get through the rima glottidis, the whole of the trachea (tracheoscopy) and of the right and left bronchus (bronchoscopy) can be directly inspected. Moreover, Killian has devised slender tubular forceps and blunt hooklets, which can be passed down the inspection tubes so as to seize and remove foreign bodies that may have become impacted in the air passages.

[1] *Brit. M.J.*, 1902, ii. 569.

**Bromide of ethyl anæsthesia.** As a general anæsthetic for operations on the nose and throat, which affords a longer period of insensibility than nitrous oxide, while avoiding the dangers and inconvenience of ether or chloroform, anæsthetics which are especially undesirable in procedures involving considerable hemorrhage in the upper respiratory tract, **bromide of ethyl** is worthy of a more extended trial than has yet been made of it in this country. In America and in France it has been largely used by laryngologists, and the favourable experience of Brown Kelly[1] with this anæsthetic will lead others to use it in suitable conditions. He reminds us that it was discovered so long ago as 1827 by Serullas, and was first used as an anæsthetic by Nunneley, of Leeds, in 1849. Kelly states that the chief characteristic of bromide of ethyl as an anæsthetic is its strikingly rapid yet transient action. A suitable dose in about a minute produces unconsciousness, lasting on an average one to two minutes, from which the patient awakes and regains almost at once his normal state. But it is very important to use only unadulterated bromide of ethyl, which is transparent and colourless, with an agreeabie chloroform-like odour ; but exposure to light and damp is prone to cause it to decompose. Being a vaso-dilator, and having no tendency to produce syncope, it may be administered with the patient seated; but like nitrous-oxide bromide, ethyl anæsthesia is often accompanied by spasmodic closure of the jaws, and therefore a mouth-gag should be placed in position before its administration. A suitable dose (for children under three years of age average one-and-a-half drachms ; between three and twelve years, two to two-and-a-half drachms; young adults, two-and-a-half to three-and-a-half drachms) should be measured out, and the whole quantity poured upon a folded piece of lint, which must be closely applied to the mouth and nostrils so that air does not enter at the sides. A slight struggling often occurs after the first few inspirations. In about one minute, more or less, the breathing becomes stertorous, and the patient should be ready for operation. In some patients who breathe slowly, or who after a few respirations cease breathing, it may be necessary to administcr the anæsthetic for a slightly longer period, or to give a breath of air, or to otherwise encourage more free respiration. Kelly states that the face becomes congested, the eyes remain open, the conjunctivæ injected, and the pupils widely dilated. In adults, excitement caused by the drug is an objection, and is sometimes considerable and even violent during the brief period of recovering consciousness. Vomiting occurs in a large proportion of patients, sometimes during, more usually after, the period of anæsthesia. Several deaths have occurred from its use, and the respirations should always be watched during administration. The drug is not recommended for prolonged anæsthesia.

[1] *Ibid*, 588.

**Measurements for operating distances in the nose.** The difficulty of determining the distances from the nasal orifices of the various intranasal structures and ostia is often a matter of serious moment, especially when the nasal passages are more or less occluded by pathological conditions for the relief of which operative treatment is essential. Mosher[1] has done useful work by measuring these distances in fifty-four half-heads (representing at least fifty different heads) in order to get an idea of the operating distances in the nose, the measurements being made on wet specimens. He gives three figures for each measurement, the first in the table appended being the measurement occurring the greater number of times (not the mathematical average), the second and third rows being the maximum and minimum measurements. In all cases the measurements were made from the lower edge of the posterior rim of the nasal opening; vertical measurements were made close to the junction of the septum and the cribriform plate.

| | Inches. | | |
|---|---|---|---|
| To the upper end of the infundibulum ... | 2 | $2\frac{1}{4}$ | $1\frac{3}{4}$ |
| ,, ostium sphenoidale ... ... ... ... ... | $2\frac{1}{2}$ | $2\frac{3}{4}$ | $2\frac{1}{4}$ |
| ,, posterior wall of the sphenoidal sinus | 3 | $3\frac{3}{4}$ | $2\frac{1}{2}$ |
| ,, lower border of the anterior wall of the sphenoidal sinus ... ... | $2\frac{1}{4}$ | $2\frac{5}{8}$ | 2 |
| ,, ostium maxillare ... ... ... ... ... | $1\frac{1}{2}$ | $1\frac{3}{4}$ | $1\frac{1}{4}$ |
| From the upper end of the infundibulum horizontally forwards to the root of the nose ... ... ... ... | 1 | $\frac{3}{8}$ | $\frac{1}{2}$ |

From measurements on the cleaned skull and in wet specimens, it appears that in wet specimens and on the living one-eighth to one-quarter of an inch has to be added to the measurements made on the cleaned skull.

The lower opening of the lachrymal duct beneath the inferior turbinate is about two-thirds of the way to the opening of the antrum. This makes the average distance one inch.

Another point of considerable importance was noted; viz., that if a burr is entered at the upper end of the infundibulum and passed directly upward in order to open the frontal sinus, it will in the majority of cases enter the cranial cavity. This, Mosher states, is due to the frequent sharp slope forward of the posterior wall of the sinus. In order to enter the sinus from this point, the burr should point forward at an angle of forty-five degrees, and would have a working distance of half an inch. In order to bore straight up into the sinus, the probe must be held parallel with the teeth and close to them.

Again, it was found that in a majority of the specimens examined, a line passed one-quarter of an inch above the

[1] *Ann. Surg.*, 1902, xxxvi. 554.

lower rim of the orbit, and carried horizontally backward, passed through the ostium maxillare; a line one-quarter of an inch below the rim cut the lower opening of the lachrymal duct; a line one-half of an inch below the rim passed through the upper opening of the Eustachian tube. Fifty cleaned skulls gave the level of the cribriform plate as the mid-point of the inner wall of the orbit, and wet specimens showed that this point is one-eighth of an inch above the inner canthus of the eye.

P. WATSON WILLIAMS.

